# Distribution of macrophages in the developing cochlea of the common marmoset, a primate model animal

**DOI:** 10.3389/fimmu.2023.1229414

**Published:** 2023-08-22

**Authors:** Makoto Hosoya, Tsubasa Kitama, Marie N. Shimanuki, Takanori Nishiyama, Naoki Oishi, Hideyuki Okano, Hiroyuki Ozawa

**Affiliations:** ^1^ Department of Otorhinolaryngology, Head and Neck Surgery, Keio University School of Medicine, Tokyo, Japan; ^2^ Department of Physiology, Keio University School of Medicine, Tokyo, Japan; ^3^ Laboratory for Marmoset Neural Architecture, Center for Brain Science, RIKEN, Saitama, Japan

**Keywords:** cochlea development, macrophages, marmoset, primate model, immune cells

## Abstract

**Introduction:**

Macrophages are essential immune cells in the cochlea that contribute to inflammation, tissue repair, and homeostasis. They also play an important role in local cochlear immunity. The developmental immigration and maturation of macrophages in the cochlea have been investigated and are considered essential for normal hearing acquisition. Most of our current knowledge regarding cochlear development is based on rodent models because of the ethical challenges of using human fetal samples for research. However, inter-species differences between rodents and humans have been reported. In this study, we used a primate animal model to investigate the distributions of macrophages in the developing cochlea. The common marmoset (*Callithrix jacchus*), a small monkey species that inhabits the New World, was used as the model.

**Methods:**

We investigated the distribution of macrophages in the developing cochlea of the common marmoset by performing immunohistochemical analyses of cochlear tissue from common marmoset embryos at different development stages.

**Results:**

We revealed detailed distribution changes in the macrophages of a primate animal model cochlea. This observation indicates that most of the changes in the general distribution of macrophages were well preserved between rodents and this primate. The distribution changes observed in the common marmoset were also compatible with observations in the human fetus; although, observations in the human fetus are limited. Our observations in this study also revealed several differences between common marmosets and rodents.

**Conclusion:**

The time course of immunological development and maturations established in this study will aid in the study of the primate-specific developmental biology of the inner ear. These observations may eventually lead to new therapeutic strategies for hearing loss in humans. In addition, understanding the immunological steady-state of the cochlea may help in the study of age- and genetic-induced hearing loss and in the design of regenerative therapies.

## 1 Introduction

The inner ear is a sensory organ essential for hearing, balance, and the perception of acceleration. The inner ear of mammals comprises several parts, and the cochlea acts as an organ for hearing perception. In the cochlea, the vibrations produced by sound waves are converted into electrical pulses in hair cells. This mechanoelectrical transmission is essential for hearing, and the electrical pulses generated in these steps are perceived in the brain as sound.

Immunological studies of the cochlea have received considerable attention, especially from the viewpoint of hearing disorders mediated by ototoxic drugs (1, 2) and noise-induced hearing loss (3–5). Moreover, a relationship between the immunological state of the cochlea and age-related hearing loss has been suggested (6, 7). There are several cells that plays immunologically important roles in the cochlea including T cells, B cells, and neutrophils. Between these cells, macrophages are the most abundant and thought to be essential immune cells in the cochlea (8). Macrophages contribute to inflammation, tissue repair, and homeostasis and are critical in the cochlea (9). In response to acute cochlear damage, macrophages will significantly modify their distribution, phenotype, and number. This observation supports the hypothesis that macrophages are vital in the cochlear response to acoustic trauma. Macrophages prevent the loss of spiral ganglion neurons after cochlear injury (10). Moreover, macrophages promote the repair of inner hair cell ribbon synapses after noise-induced cochlear synaptopathy (11).

The immigration and maturation of macrophages in the cochlea is essential for normal hearing (12–15). Most studies investigated rodent models because of restrictions on the use of human fetal temporal bone samples, and only a few reports have addressed human cochlear macrophage development (16, 17). In addition, cochlear development in humans is not fully understood because it occurs in the late phases of gestation (18–20). Moreover, ethical regulations have prevented the study of late-stage fetal human samples. As yet, several inter-species differences in the cochlear development of primates and rodents have been reported. Therefore, it has been suggested that our knowledge based on rodent models cannot always be directly applied to humans (19). To overcome this the common marmoset (*Callithrix jacchus*), a small primate, has been used to understand cochlear development(21–25), as well as genetic and age-related hearing loss (26). A similar approach has been applied in investigations on the brain (27).

We previously reported on cochlear development in the common marmoset, highlighting basic anatomical staging of the developing cochlea, and comparisons with that in humans and mice. We further revealed several differences between rodents and marmosets in the development of the organ of Corti, spiral ganglion neurons, and the stria vascularis. Moreover, we demonstrated similarities with human developmental processes, indicating that this primate is a promising animal model (21, 22, 24). Nevertheless, researchers have not yet examined the cochlear development of the common marmoset from an immunological viewpoint.

In this study, the development of the cochlea was investigated from an immunological viewpoint, and the distribution of macrophages in the developing cochlea of a non-human primate animal model was examined. The present study provides valuable insights on the process of macrophage invasion and the role of macrophages in cochlear development in this animal model. This study aims to contribute to the development of feasible treatment methods to modulate macrophage activity in sensorineural hearing loss or hearing loss caused by noise or ototoxic agents.

## 2 Materials and methods

Cadaverous temporal bone samples from common marmoset fetuses at E70 (n = 4), E77 (n = 2), E87 (n = 4), E96 (n = 3), E109 (n = 3), E120 (n = 3), P0 (n = 3), and young adults (n = 3) were used in this study, as described in our previous reports (21, 22, 24). Adult animals were anesthetized via isoflurane inhalation (1.5–4%). Caesarion section was performed as previously described (28). Embryos were anesthetized on ice and deeply dissected in phosphate-buffered saline (PBS). Finally, the temporal bones containing cochlea were removed from the skull. Part of the tissue samples in this study were those prepared and used in our previous studies. Animal experiments were approved by the Animal Experiment Committee of Keio University (approval numbers: 11006, 08020). All animal experiments were performed in accordance with the guidelines of the National Institutes of Health and the Ministry of Education, Culture, Sports, Science, and Technology of Japan.

### 2.1 Tissue preparation

The temporal bones were dissected immediately after euthanasia. The samples were fixed overnight in 4% paraformaldehyde in PBS overnight. The specimens were embedded in Tissue-Tek O.C.T. compound (Sakura Fine Technical Co., Ltd., Tokyo, Japan) for cross-sectioning. P0 specimens were embedded in Tissue-Tek O.C.T. compound after decalcification in Decalcifying Solution B (Wako, Osaka, Japan) for one week, as described in our previous reports (21, 24, 29). Sections (7 µm) were used for immunohistochemical analysis.

### 2.2 Antibodies

The following primary antibodies were used in this study: Anti-ionized calcium binding adapter molecule 1 (IBA1; Rabbit IgG; ab5076, Abcam, Cambridge, UK, 1:500), anti-POU domain class 3 transcription factor 4 (POU3F4; HPA; HPA031984, Atlas Antibodies, Bromma, Sweden; 1:500), anti-SRY-box transcription factor 10 (SOX10; Mouse IgG1; 365692, Santa Cruz Biotechnology, Santa Cruz, CA, USA; 1:200), anti-cochlin (COCH; Rabbit IgG; HPA050122, Atlas Antibodies; 1:200), anti-SRY-box transcription factor (SOX2; Rabbit IgG; ab92494, Abcam; 1:200), anti- myosin 7A (MYO7A; Mouse IgG1; 138-1-s, DSHB, GeneTex, Irvine, CA, USA; 1:30), anti-MYO7A (Rabbit IgG; 25-6790, Proteus Biosciences, Ramona, CA, USA; 1:500), anti-human leukocyte antigen (HLA) DQ/DR/DP (Mouse IgG1; CR3/43, NB120-17101, Novus, St. Charies, MO, USA; 1:50), anti-neurofilament heavy chain (NEFH; Chick IgY; ab4680, Abcam, 1:1000), anti-S100 calcium-binding protein B (S100B; Rabbit IgG; ab52642, Abcam; 1:200), anti-myelin protein zero (MPZ; Rabbit IgG; ab31851, Abcam, Cambridge, UK; 1:200), anti-tubulin beta 3 (TUBB3; Mouse IgG2b; GTX631836, DSHB, GeneTex, 1:1000), anti-melan A (MLANA; Rabbit IgG; NBP1-30151, Novus; 1:500), anti-sodium/potassium-transporting ATPase subunit alpha-1 (ATP1A1; Mouse IgG2a; a6F, DSHB, Iowa City, IA, USA; 1:500), anti-actin alpha 2 (ACTA2; Mouse IgG2a; A2547, SIGMA, Saint Louis, MO, USA; 1:200), anti-solute carrier family 2 member 1 (SLC2A; Rabbit IgG; ab115730, Abcam; 1:500), anti-cluster of differentiation 163 (CD163; Rabbit IgG; ab182522, Abcam; 1:200).

The following secondary antibodies were used in this study: Donkey anti-goat IgG (Alexa Fluor Plus 647; A32814, Invitrogen, Waltham, MA, USA; 1:500), donkey anti-rabbit IgG (Alexa Fluor Plus 555; A32794, Invitrogen; 1:500), donkey anti-rabbit IgG, (Fluor Plus 647; A32795, Invitrogen; 1:500), donkey anti-mouse IgG (Alexa Fluor Plus 555; A32773, Invitrogen; 1:500), donkey anti-mouse IgG (Alexa Fluor Plus 647; A32787, Invitrogen; 1:500), and donkey anti-chicken IgY (Alexa Fluor 647; 703-605-155, Jackson Immuno-Research, West Grove, PA, USA; 1:500).

### 2.3 Immunohistochemistry

The tissue sections were heated (80°C) in 10 µM citrate buffer (pH 6) for 15 min, after a brief wash with PBS. Then, the sections were pre-blocked in PBS containing 10% normal serum for one hour at room temperature. Next, the sections were incubated with the relevant primary antibodies overnight at 4°C. Finally, the sections were incubated with Alexa Fluor-conjugated secondary antibodies for 60 min at room temperature. The nuclei were counterstained with Hoechst 33258.

## 3 Results

### 3.1 Distributions of macrophages in the developing cochlea of the common marmoset

**Figure 1:**
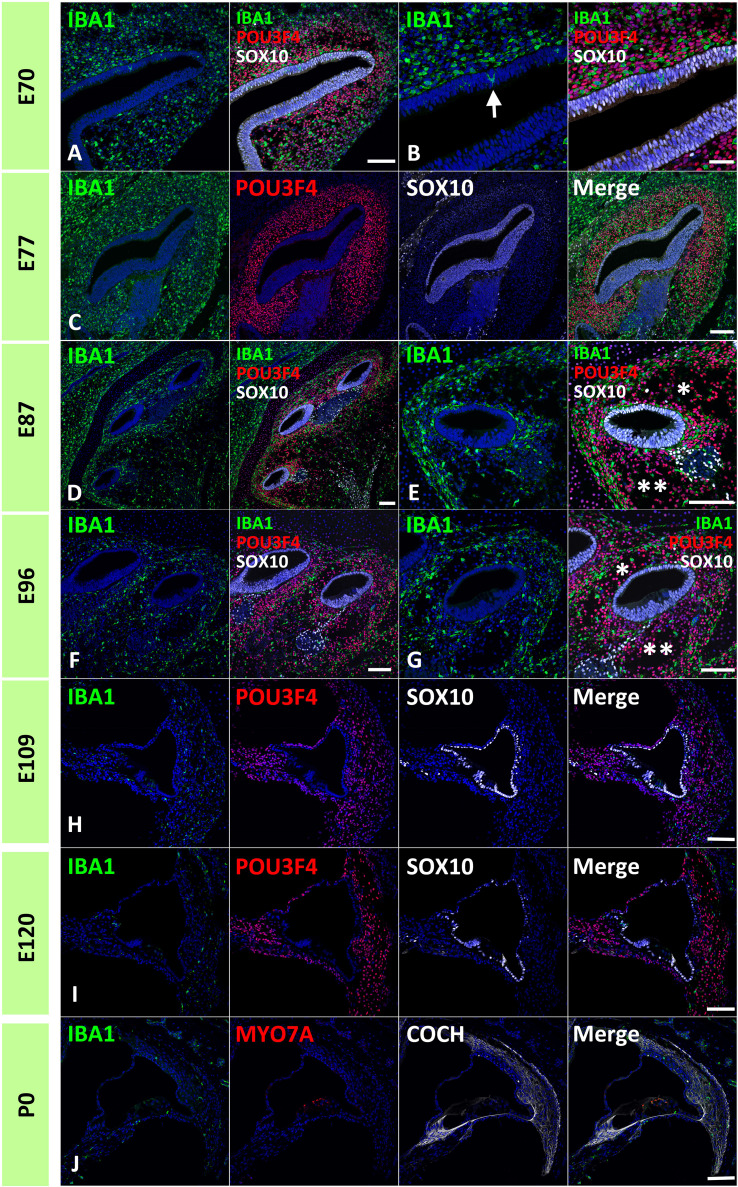
Distribution of ionized calcium-binding adapter molecule 1 (IBA1)-positive cells in the developing primate cochlea. **(A, B)** Expression of IBA1 in E70 cochlea. IBA1-positive cells are broadly distributed surrounding the elongating SRY-box transcription factor 10 (SOX10)-positive cochlear duct. IBA1-positive cells distributed among POU domain class 3 transcription factor 4 (POU3F4)-positive periotic mesenchymal cells. Several IBA1-positive cells infiltrate into the elongated SOX10-positive cochlear duct at this stage (Arrow in **B**). **(C)** Expression of IBA1 in the E77 cochlea. At this stage, IBA1-positive cells are broadly observed surrounding SOX10-positive cells and among the POU3F4-positive periotic mesenchymal cells. **(D, E)** Expression of IBA1 in the E87 cochlea. At this stage, numerous IBA1-positive cells are observed surrounding cochlear duct. Compared to early stages, IBA1-positive cell distribution is heterogeneous. Near the cochlear duct, IBA1-positive cells are abundantly observed in the presumptive lateral wall fibrocytes aria, whereas only a few IBA1-positive cells are observed in presumptive scala vestibuli (* in **E**) and scala tympani (** in **E**). **(F, G)** Expression of IBA1 in E96 cochlea. At this stage, relatively abundant IBA1-positive cells are still observed in the lateral wall fibrocytes’ aria. In contrast, only a few IBA1-positive cells are observed in presumptive scala vestibuli (* in **G**) and scala tympani (** in **G**). **(H)** Expression of IBA1 in E109 cochlea. Compared with the early stages, there is an obvious decrease in the number of IBA1-positive cells. A small number of IBA1-positive cells are observed in the lateral wall fibrocytes. **(I, J)** Expression of IBA1 in E120 and P0 cochlea. At these stages, several IBA1-positive cells are distributed in the cochlea. A relatively large number of IBA1-positive cells are located in the spiral ligament fibrocytes; however, their numbers are less than those observed in stages before E96. Scale Bar: 100 μm in **(A, C–J)**; 50 μm in **(B)** The nuclei were counterstained with Hoechst (blue).

First, IBA1 expression was examined in the developing cochlea of the common marmoset. IBA1 encodes the ionized calcium-binding adapter molecule 1, an established macrophage marker in the cochlea of both rodents (30, 31) and humans (32–34). IBA1 has also been used as a macrophage marker in common marmosets (35). Abundant cells expressing IBA1 were observed as early as E70 surrounding the cochlear duct. At this stage, IBA1-positive cells were localized to POU3F4-positive developing periotic mesenchymal cells (**Figures 1A, B**). Notably, several IBA1-positive cells that integrated into the cochlea were observed at this stage (indicated by arrows in **Figure 1B**). At E77 and E87, abundant IBA1-positive cells surrounded the SOX10-positive cochlear epithelium and were present among periotic mesenchymal cells (**Figures 1C–E**). At E96, IBA1-positive cells were observed mainly among periotic mesenchymal cells, especially in the fibrocytes of the developing lateral wall (**Figures 1F, G**). As late as E96, relatively abundant IBA1-positive cells were observed in the developing cochlea. After E109, the number of IBA1-positive cells gradually decreased; especially between E96 and E109, the decrease in IBA1-positive cells was prominent (**Figures 1H, I**). At P0, a small number of IBA-1 positive cells were observed in the cochlea, including lateral wall fibrocytes, stria vascularis, and organs of Corti (**Figure 1J**).

### 3.2 Distributions of macrophages in the sensory epithelium of the common marmoset

**Figure 2:**
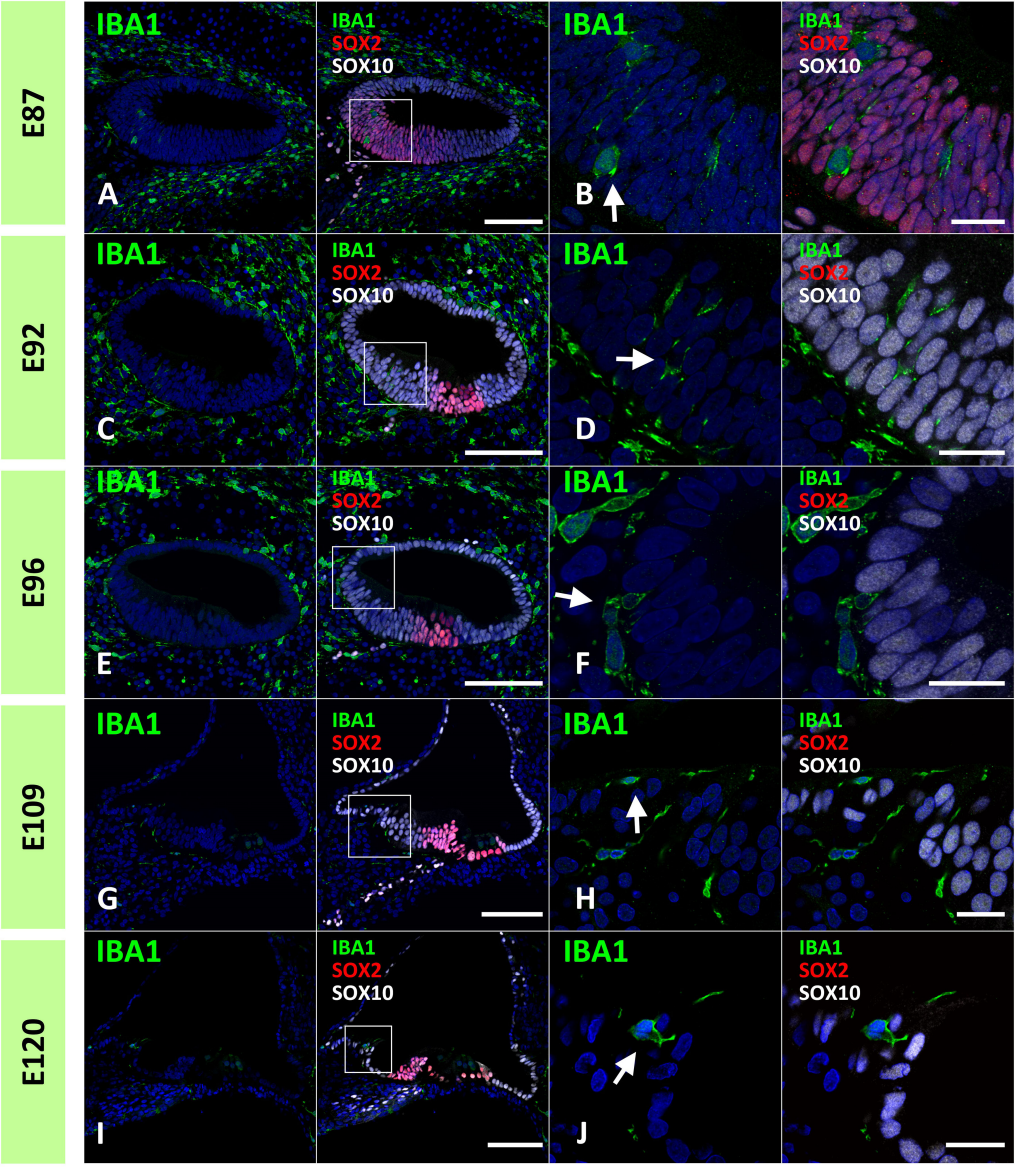
Distribution of ionized calcium-binding adapter molecule 1 (IBA1)-positive cells in the developing sensory epithelium of the primate cochlea. **(A, B)** Distribution of macrophages in E87 cochlear sensory epithelium. In this stage, a small number of the IBA1-positive cells are integrated into the SRY-box transcription factor 10 (SOX10)-positive cochlear epithelium (Arrow in **B**). **(C, D)** Distribution of macrophages in E92 cochlear sensory epithelium. IBA1-positive cells are observed in the SOX10+ and SRY-box transcription factor 2 (SOX2) negative Kölliker’s organ of the developing cochlea (Arrow in **D**). No IBA1-positive cells are observed in the SOX2-positive region. **(E, F)** Distribution of macrophages in E96 cochlear sensory epithelium. IBA1-positive cells observed in the SOX10+ and SOX2-negative Kölliker’s organ of the developing cochlea (Arrow in **F**). **(G–J)** Distribution of macrophages in E109 and E120 cochlear sensory epithelium. At E109 and E120, macrophages that integrated into the epithelium cells are observed in the epithelium covering the spiral limbus (Arrow in **H, J**). Scale Bar: 100 μm in **(A, C, E, G, I)**; and 20 μm in **(B, D, F, H, J)** The nuclei were counterstained with Hoechst (blue). High magnification images of squared area in **(A, C, E, G, I)** are shown in **(B, D, F, H, J)**, respectively.

**Figure 3:**
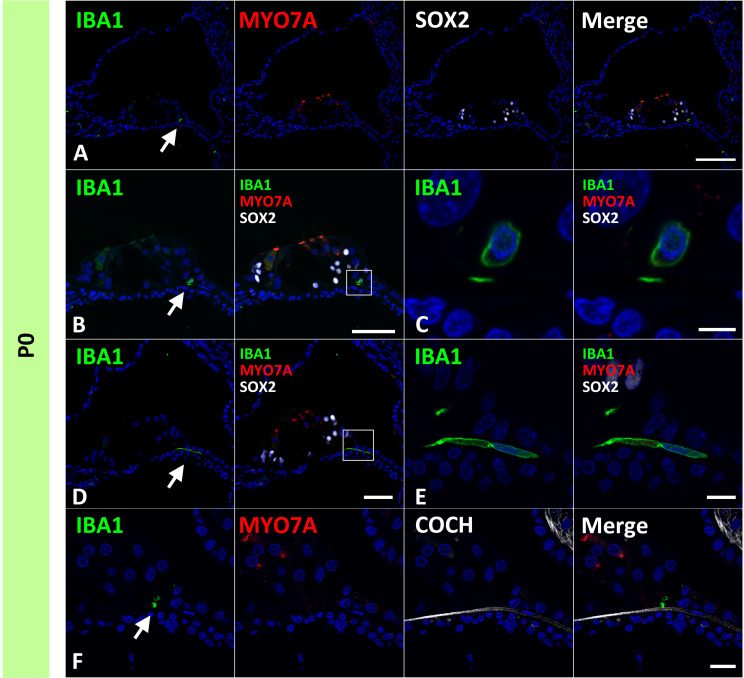
Distribution of ionized calcium-binding adapter molecule 1 (IBA1)-positive cells in the postnatal day 0 organ of Corti. **(A–F)** Expression of IBA1 in organ of Corti of P0 cochlea. In the P0 cochlea, IBA1-positive cells are observed in the organ of Corti [Arrow in **(A, B and D)**]. IBA1-positive cells are observed above cochlin (COCH)-positive basilar membrane [Arrow in **(F)**]. High magnification images of squared area of **(B, D)** are shown in **(C, E)** respectively. Scale Bar: 100 μm in **(A)** 50 μm in **(B, D)** 5 μm in **(C)**, 10 μm in **(E)** 20 μm in **(F)**. The nuclei were counterstained with Hoechst (blue).

Next, we examined the infiltration of IBA1-positive cells into the sensory epithelium of the cochlear duct. As early as E87, a small number of IBA1-positive cells were integrated into the SOX10-positive cochlear epithelium (**Figures 2A, B**). Except for infiltrations into the stria vascularis, the infiltration of macrophages was observed only in the modiolar half of the cochlear duct. After E92, macrophage infiltration was observed in SOX2-negative cells (**Figures 2C–F**). Macrophage infiltration into SOX10-positive cochlear epithelium was observed as late as E120. At E109 and E120, macrophage integration into epithelial cells was observed in the epithelium covering the spiral limbus (**Figures 2G–J**). At P0, macrophages were observed in the organ of Corti, whereas no IBA1-expression was observed until E120 in the SOX2-positive developing organ of Corti (**Figure 3**). Most macrophages in the organ of Corti were observed around Deiters’ cells and Hensen’s cells beneath the outer hair cells.

### 3.3 Distributions of macrophages in the spiral ganglion of the common marmoset

**Figure 4:**
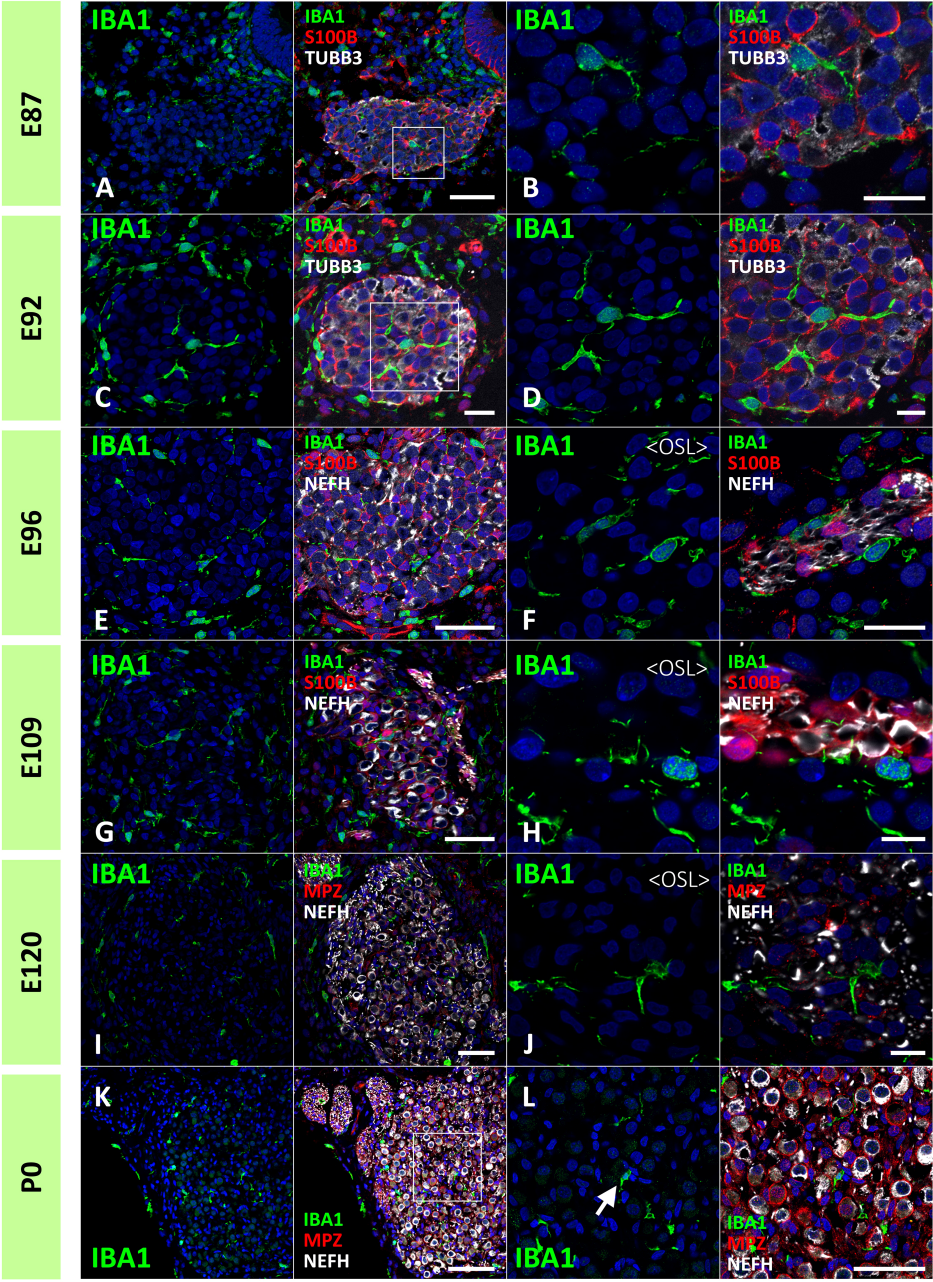
Distribution of ionized calcium-binding adapter molecule 1 (IBA1)-positive cells in the developing spiral ganglion neuron. **(A, B)** IBA1 expression in the spiral ganglion neurons of E87 cochlea. At this stage, IBA1-positive macrophage infiltration into the spiral ganglion is observed. The IBA1-positive cells were observed among the neurofilament heavy chain (NEFH)-positive neuronal cells and S100 calcium-binding protein B (S100B)-positive immature glial cells [A high magnification image of the squared area of **(A)** is shown in **(B)**]. **(C, D)** IBA1 expression in the spiral ganglion neurons of E92 cochlea. At this stage, IBA1-positive macrophages are observed among the NEFH-positive cells and S100B-positive glial cells. [High magnification image of the squared area of **(C)** is shown in **(D)**]. **(E–H)** IBA1 expression in the spiral ganglion neurons of E96 and E109 cochlea. At this stage, IBA1-positive macrophages are observed among the NEFH-positive cells and S100B-positive glial cells. Macrophages are also observed among the neurons in the osseous spiral lamina **(F, G)**. **(I, J)** IBA1 expression in the spiral ganglion neurons of E120. At this stage, IBA1-positive macrophages are observed among the NEFH-positive cells and myelin protein zero (MPZ)-positive myelinating glial cells. Macrophages are also observed among the neurons in the osseous spiral lamina. **(K, L)** IBA1 expression in the spiral ganglion neurons of P0. At this stage, IBA1-positive macrophages are observed among the NEFH-positive cells and MPZ-positive myelinating glial cells [Arrow in **(L)**]. **(A)** high magnification image of the squared area of **(K)** is shown in **(L)**. Scale Bar: 50 μm in **(A, E, G, I)** 20 μm in **(B, C, F)**; 10 μm in **(D, H, J)**; 100 μm in **(K)** The nuclei were counterstained with Hoechst (blue).

Next, the localization of macrophages in the ganglion neurons was examined. As early as E87, macrophages infiltrated spiral ganglion neurons (**Figures 4A, B**). Throughout the development of spiral ganglion neurons, infiltrating macrophages were observed among NEFH-positive spiral ganglion neurons and S100B-positive developing glial cells (**Figures 4C–H**). Macrophage infiltration into spiral ganglion neurons was also observed at the time of MPZ-positive myelin formation in glial cells (**Figures 4I, J**). Macrophages were also observed among the neurons in the osseous spiral lamina as late as E96 (**Figures 4F, H, J**). At P0, Macrophages were observed in the spiral ganglion located between the NEFH-positive neural cells and MPZ-positive glial cells (**Figures 4K, L**).

### 3.4 Distributions of macrophages in the stria vascularis of the common marmoset

**Figure 5:**
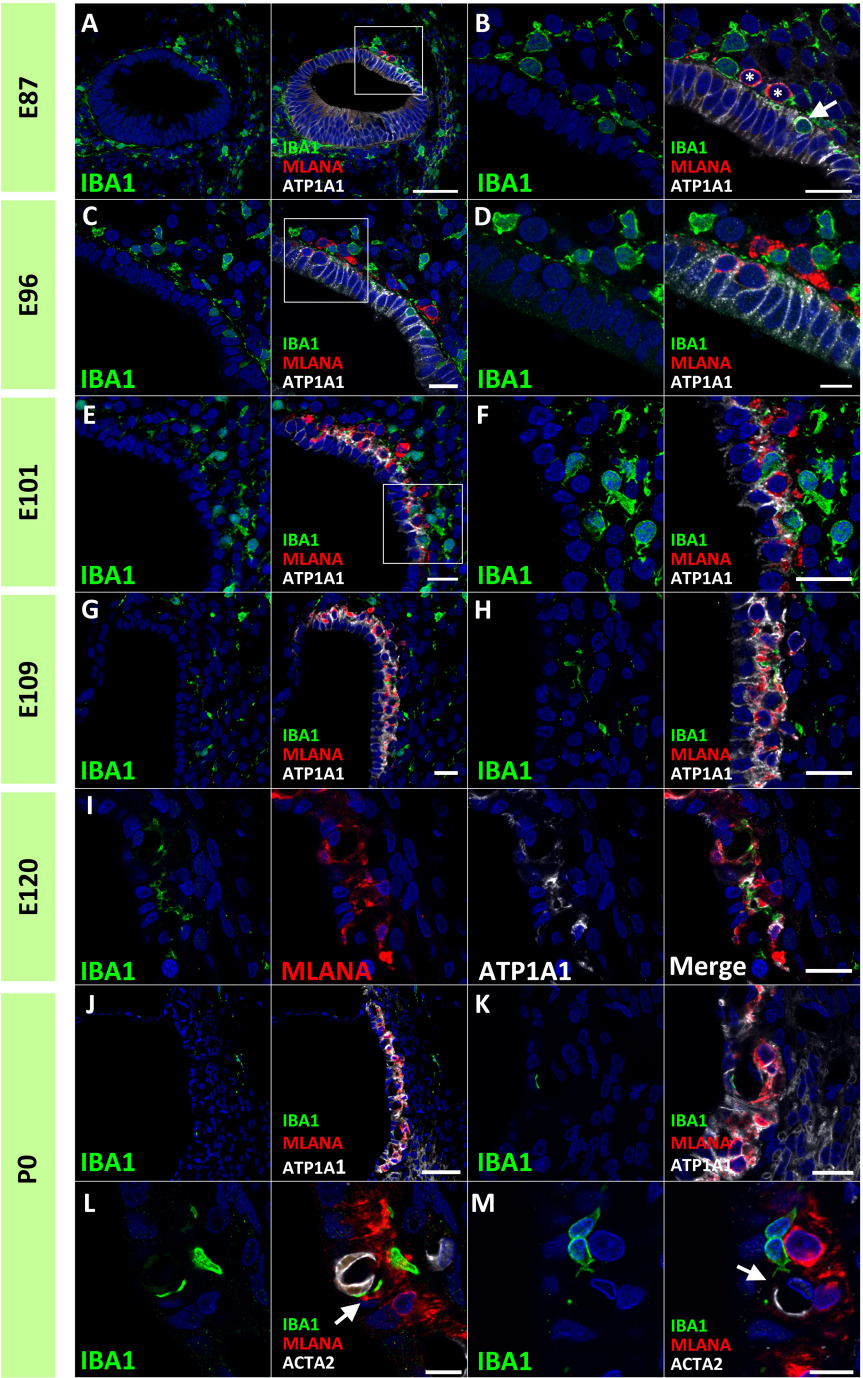
Distributions of ionized calcium-binding adapter molecule 1 (IBA1)-positive cells in the developing stria vascularis. **(A, B)** IBA1 expression in the stria vascularis of E87 cochlea. At E87, infiltration of IBA1-positive cells [Arrow in **(B)**] between sodium/potassium-transporting ATPase subunit alpha-1 (ATP1A1)-positive cochlear epithelium is observed, whereas melanoma antigen recognized by T cells (MLANA)-positive cells are located just next to marginal cells [* in **(B) (A)** high magnification image of the squared area of A is shown in **(B)**]. **(C, D)** IBA1 expression in the stria vascularis of E96 cochlea. Both IBA1-positive cells and MLANA-positive cells are integrated between the ATP1A1-positive marginal cells [**(A)** high magnification image of the squared area of **(C)** is shown in **(D)**]. **(E, F)** IBA1 expression in the stria vascularis of E101 cochlea. IBA1-positive cells, MLANA-positive cells and marginal cells formed complicated cell-cell contact at this stage [**(A)** high magnification image of the squared area of **(E)** is shown in **(F)**]. **(G–I)** IBA1 expression in the stria vascularis of E109 **(G, H)** and E120 cochlea **(I)**. At E109 and E120, IBA1-positive cells decrease from the incorporated space between the marginal cells and intermediate cells. **(J–M)** IBA1 expression in the stria vascularis of P0 cochlea. At P0, IBA1-positive cells were observed in perivascular spaces as perivascular macrophages (PVMs) among the incorporated space between the marginal and intermediate cells. No melanoma antigen recognized by T cells (MLANA) expression was observed in IBA1-positive cells. IBA1-positive cells lapped the actin alpha 2 (ACTA2)-positive capillaries in the stria vascularis [Arrows in **(L, M)**] [**(L)**, another image is shown in **(M)**]. Scale Bar: 50 μm in **(A)**, and **(J)** 20 μm in **(B, C, E–I, K)**; 10 μm in **(D, L, M)**. The nuclei were counterstained with Hoechst (blue).

**Figure 6:**
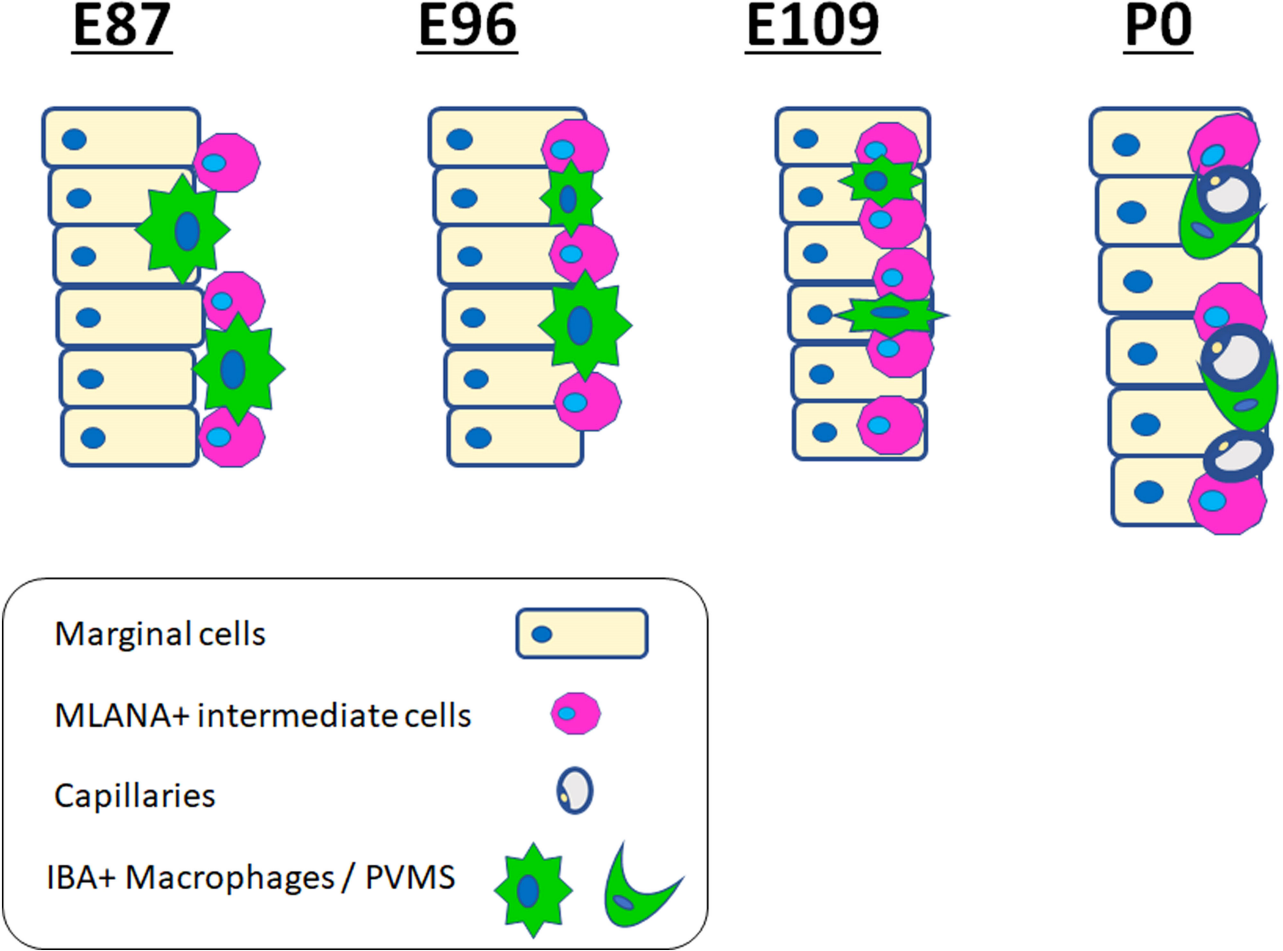
Schematic diagrams of distribution of ionized calcium-binding adapter molecule 1 (IBA1)-positive cells in the stria vascularis. Schematic diagrams showing the time course of IBA1-positive cell and MLANA-positive cell infiltration into the stria vascularis. IBA1-positive cell infiltrations can be observed, proceeding to MLANA-positive cell infiltration. Then, as development proceeds, IBA1-positive cells decrease and are finally observed only as PVMs lapping the capillaries.

Next, we examined macrophage infiltration into the stria vascularis. Infiltration of intermediate cells marked with MLANA into marginal cells marked with ATP1A1 has been reported in the developing stria vascularis (24). In this study, we analyzed the migration of IBA1-positive macrophages by comparing intermediate and marginal cell development. At E87, the infiltration of IBA1-positive cells between ATP1A1-positive cochlear epithelia was observed (**Figures 5A, B**). In contrast, as previously reported (24), no MLANA-positive cell infiltration was detected; MLANA-positive cells were located next to the marginal cells. At E96, both intermediate cells and macrophages were integrated between ATP1A1-positive marginal cells (**Figures 5C, D**). At E101, when the marginal and intermediate cells formed complicated cell-cell contacts, macrophages were also embedded between these cells (**Figures 5E, F**). At E109 and E120, a decrease in the number of macrophages between the incorporated marginal and intermediate cells was observed, whereas small numbers of macrophages were observed in this space (**Figures 5G–I**). At P0, only a few perivascular macrophages (PVMs) were located in the perivascular spaces (**Figures 5J–M**), as previously reported in rodents (36). At this stage, PVMs had arms that held small vessels in the stria vascularis. A schematic representation of macrophage infiltration into the stria vascularis is shown in **Figure 6**.

### 3.5 Localization of macrophage subpopulations in the developing cochlea of the common marmoset

Next, to estimate the localization of macrophage subpopulations in the developing primate cochlea, the expression of CD163 was examined. CD163 is a protein encoded by the *CD163* gene. CD163 is a high-affinity scavenger receptor for the hemoglobin-haptoglobin complex (37). It is also a marker of cells of the monocyte/macrophage lineage and acts as an innate immune sensor for Gram-positive and Gram-negative bacteria. CD163 expression in the developing and adult cochlea in humans has been previously reported (17, 32, 33, 38).

**Figure 7:**
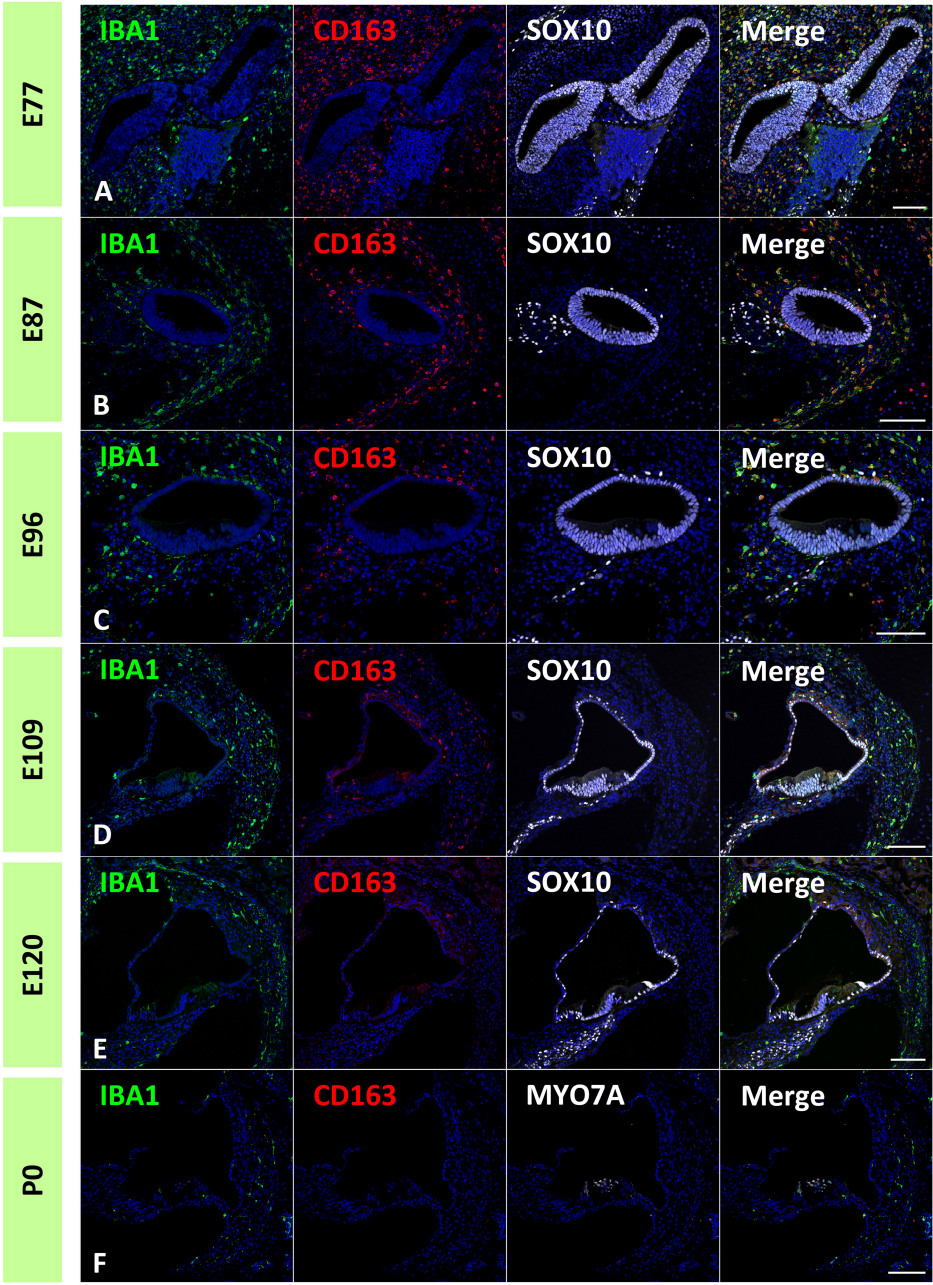
Distribution of cluster of differentiation 163 (CD163)-positive cells in the developing cochlea. **(A)** CD163 expression in the E77 cochlea. Abundant CD163-positive cells were observed among the periotic mesenchymal cells, and most ionized calcium-binding adapter molecule 1 (IBA1)-positive cells also expressed CD163. **(B)** CD163 expression in the E87 cochlea. CD163-and IBA1-double positive cells are observed broadly in periotic mesenchymal cells. **(C, D)** CD163 expression in the E96 and E109. CD163 expression in the IBA1-positive cells gradually decreased with development. **(E)** CD163 expression in the E120 cochlea. Only a few IBA1-positive cells show CD163 expression. **(F)** CD163 expression in P0 cochlea. At P0, no CD163 expression is detected in the sensory epithelium. Scale Bar: 100 μm. The nuclei are counterstained with Hoechst (blue).

**Figure 8:**
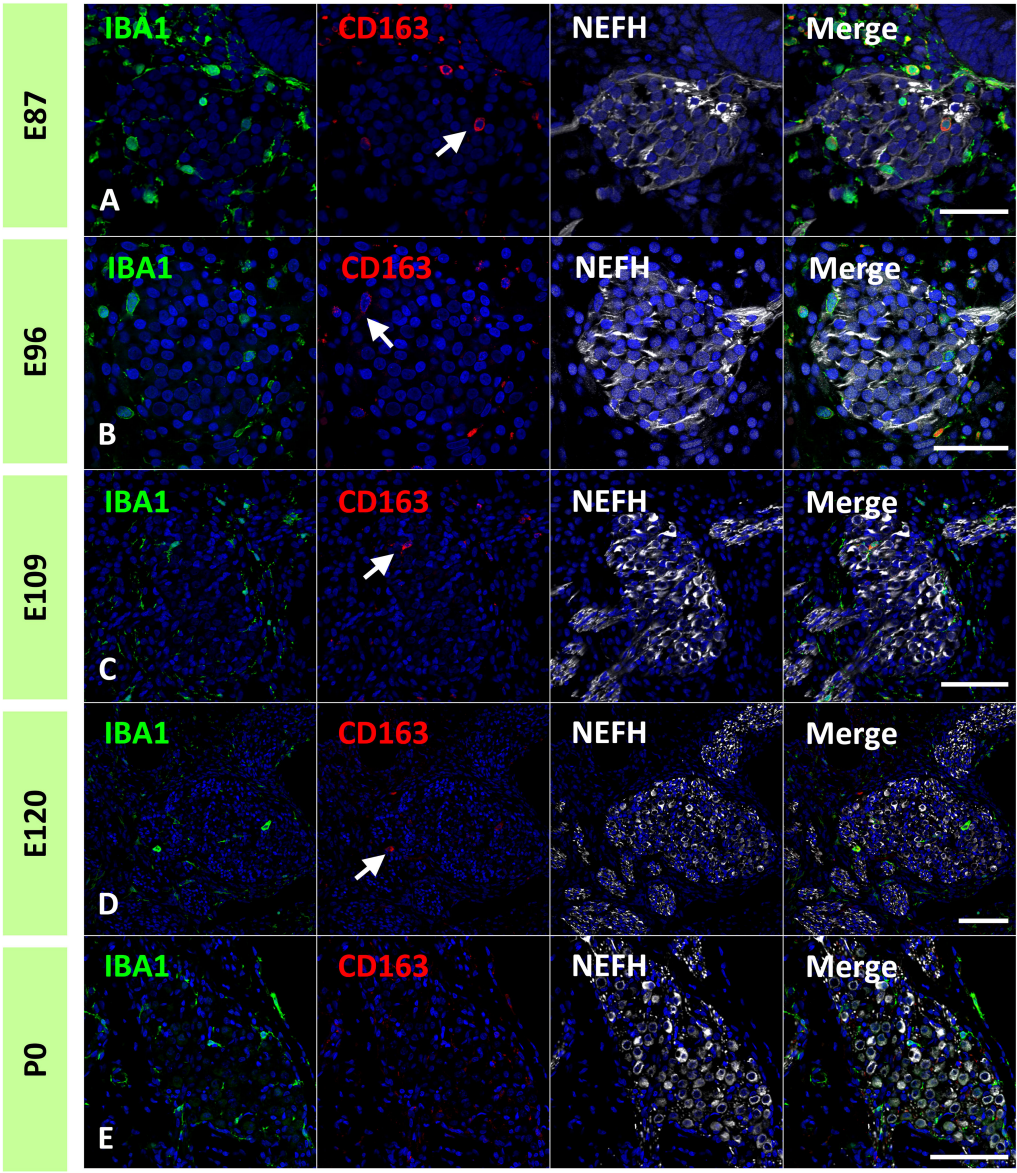
Expression of cluster of differentiation 163 (CD163) cells in the developing spiral ganglion neuron. **(A–D)** CD163 expression in the E87, E96, E109, and E120 spiral ganglion neurons. In the developing spiral ganglion, some ionized calcium-binding adapter molecule 1 (IBA1)-positive cells show CD163 expression until E120 [Arrows in **(A–D)**]. **(E)** CD163 expression in the P0 spiral ganglion neurons. No CD163 expression could be detected at this stage. Scale Bar: 50 μm in **(A, B)** 100 μm in **(C–E)**. The nuclei are counterstained with Hoechst (blue).

In the common marmoset, CD163 expression in macrophages was observed as early as E77 (**Figure 7A**). At this stage, abundant CD163-positive cells were observed among periotic mesenchymal cells, and most IBA1-positive cells expressed CD163. After E87, a gradual decrease in CD163-positive macrophages was observed (**Figures 7B–D**). At E120, only a few IBA1-positive cells expressed CD163 (**Figure 7E**). At P0, no CD163 expression was detected in the sensory epithelium (**Figure 7F**). The distribution of CD163-positive cells in spiral ganglion neurons was also examined. In the developing spiral ganglion, some IBA1-positive cells exhibited CD163 expression as early as E87 (**Figure 8A**). Some macrophages in the spiral ganglion continued to express CD163 expression until E120 (**Figures 8B–D**), whereas no CD163 expression was detected in the P0 spiral ganglion (**Figure 8E**).

### 3.6 Expression patterns of Class II MHC components in the developing cochlea of the common marmoset

**Figure 9:**
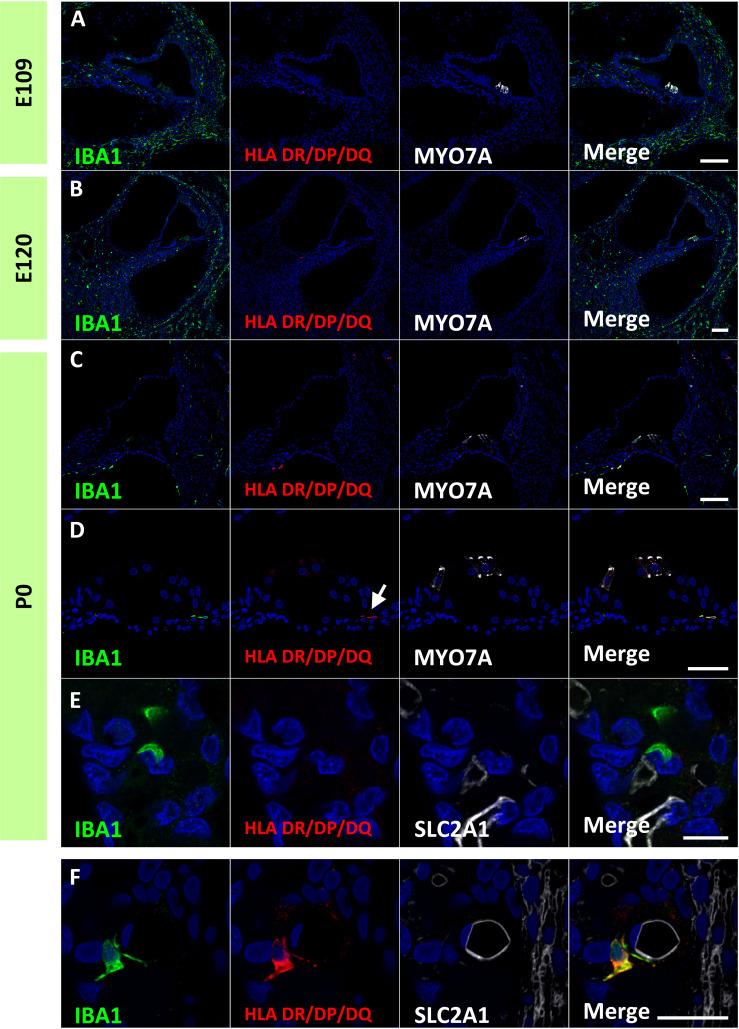
Expression of human leukocyte antigen (HLA) DR/DP/DQ in the developing cochlea. **(A, B)** Expression of HLA DR/DP/DQ in the E109 and E120 cochlea. No HLA DR/DP/DQ expression is observed in these stages. **(C–E)** Expression of HLA DR/DP/DQ in the P0 cochlea. At this stage, some macrophages expressed HLA DR/DP/DQ. HLA DR/DP/DQ expression is observed in macrophages in the organ of Corti [Arrow in **(D)**]. No HLA DR/DP/DQ expression is detected in the perivascular macrophages (PVMs) in the stria vascularis at this stage **(E)**. **(F)** HLA DR/DP/DQ expression in the postnatal stria vascularis. HLA DR/DP/DQ expression is detected in the postnatal PVMs. Scale Bar: 100 μm in **(A–C)** 50 μm in **(D, E)** 20 μm in **(F)** The nuclei are counterstained with Hoechst (blue).

Finally, expression patterns of class II major histocompatibility complex (MHC) components were investigated. Class II MHC is a characteristic marker of macrophages, which is essential for antigen presentation and functions as a key regulator of adaptive immune responses (39). MHC class II proteins are expressed in macrophages, B cells, monocytes, and dendritic cells and comprises HLA DR/DP/DQ. In the developing cochlea of the common marmoset, HLA DR/DP/DQ expression was not observed in macrophages in the developing cochlea as late as E120 (**Figures 9A, B**). At P0, some macrophages expressed HLA-DR/DP/DQ (**Figure 9C**), including macrophages in the organs of Corti (**Figure 9D**). In contrast, no HLA DR/DP/DQ expression was observed in the PVMs of the stria vascularis at this stage (**Figure 9E**). However, HLA DR/DP/DQ expression in PVMs was detected in the postnatal stages (**Figure 9F**), as reported in human PVMs (33).

## 4 Discussion

**Figure 10:**
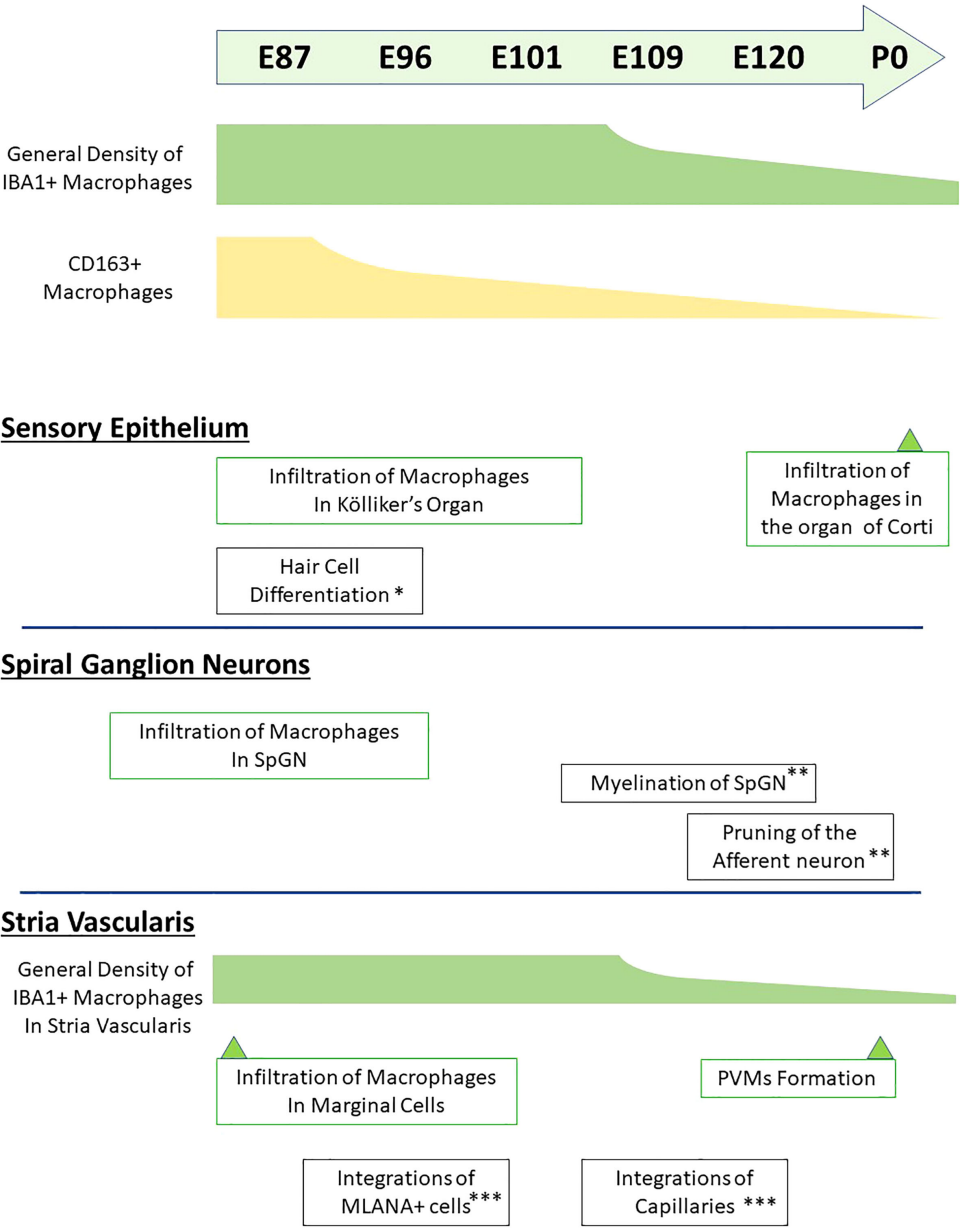
Timeline of macrophage distribution during common marmoset cochlear development. Timeline of developmental distribution of macrophage is shown in the schema comparing other important cochlear developmental steps. (*Reference: 21; **Reference: 22; ***Reference: 24). IBA1+, ionized calcium-binding adapter molecule 1-positive; CD163+, cluster of differentiation 163-positive; SpGN, spiral ganglion neurons; PVMs, perivascular macrophages; MLANA+, melanoma antigen recognized by T cells-positive.

To the best of our knowledge, this is the first report describing macrophage distribution in the developing cochlea of a primate animal model (**Figure 10**). A previous study showed dynamic changes in the distribution of resident macrophages depending on the cochlear tissue area or developmental stage (13, 14), in mice as well as in humans (17). In mice, the density of cochlear macrophages increases as the mice grow, it peaks around the neonatal stages, and decreases from P3 onward (14). Similar dynamic changes were also observed in the developing cochlea of the common marmoset, although its peak was observed around E77–87 and decreases in the macrophages in the cochlea were prominent after E109 (**Figure 1**). Combined with a previous observation that P0 in mice is the morphological equivalent of E101 in the marmoset (21), changes in the distribution of macrophages in the common marmoset and rodents are conserved, except for the relative timing of birth.

After birth, the immunological circumstances rapidly change. The outer and middle ears are not sterile at the time of birth, and these changes cannot be underestimated in the immunological state of the inner ear. However, in primates, our observations indicate that changes in the distribution of macrophages do not depend on delivery. This preservation also indicates that developmental rather than environmental changes control the changes in immunological conditions in the cochlea.

While it has been reported that the mature organ of Corti in mice lacks tissue macrophages (3, 30, 40), the developing postnatal organ of Corti contains macrophages (13). In rodents, macrophages were transiently observed beneath the organ of Corti around postnatal day 7 (13). Likewise, in the present study, we observed macrophages beneath the organ of Corti at P0, although in early stages of development, no infiltration of macrophages was observed in SOX2-positive regions, except at E87 when broad SOX2 expression was observed (**Figures 2**, **3**). To date, no observation of macrophage infiltration of the organ of Corti in the developing human cochlea has been reported, and no infiltration of macrophages into the organ of Corti was observed in a previous report examining the cochlea of human fetuses in the 9^th^ to 17^th^ week of gestation (17). Considering our previous observation that the developing cochlea of the P0 common marmoset is equivalent to approximately 24 gestational weeks in human fetuses (21), macrophage infiltration may occur at the late stages in human fetuses. However, in most cases, late human fetal sampling is unethical. From this point of view, the common marmoset is a suitable alternative to a human fetus for studying the developmental roles of macrophages in the organs of Corti in primates.

Previous reports showed important roles of cochlear macrophages in the refinement of connections between spiral ganglion neurons and hair cells, both in normal development (13, 41) and after noise trauma (11). Based on previous observations in rodents, macrophages were suspected to facilitate the formation or pruning of cochlear ribbon synapses during cochlear development (41). Although the pruning of the spiral ganglion neurons occurs between E115 and P0 in the common marmoset (22), an increase in the number of macrophages in the spiral ganglion neuron or the osseous spiral lamina was not observed at this stage. Macrophage infiltration in the spiral ganglion neurons was observed in the early stages, and no obvious increase in spiral ganglion neurons was detected (**Figure 4**). This observation indicated a limited number of macrophages regarding this pruning in the primate cochlea. Moreover, it has been suggested that macrophages are also related to the myelination of spiral ganglion neurons in rodents (12). However, macrophages in spiral ganglion neurons in the primate cochlea are observed much earlier than those in the myelination of spiral ganglion neurons, which was first observed at E115 (22). These observations, in which the infiltration of macrophages occurred much earlier than that in neural pruning or myelination, suggest other developmental roles for cochlear macrophages in the primate cochlea. Hence, to determine the exact role of cochlear macrophages, further studies on these animals are warranted.

Several previous studies in rodents have shown macrophage infiltration into the stria vascularis and the subsequent development of these macrophages into PVMs (36, 42). Observations in the present study also demonstrated that PVMs in the stria vascularis originate from infiltrating macrophages (**Figure 5**). In addition, this study benefits from the species-specific trait that the primate cochlea develops three times slower than that of other species (21) and the detailed infiltration of macrophages can be studied by comparing the integration of MLANA-positive intermediate cells into marginal cells in the stria vascularis. In primates, macrophage integration into the stria vascularis was detected in the early phases of cochlear development, just before the integration of MLANA-positive cells was observed. As MLANA-positive cell integration proceeds, macrophage infiltration becomes obvious.

Based on a previous study using rodent models, PVMs and MLANA-positive cells were believed to have the same origin (36). However, a recent study suggested that PVMs in the stria vascularis have a different origin than melanin-positive intermediate cells (43); therefore, the relationship between PVMs and intermediate cells in rodents remains unclear (42). During the development of the stria vascularis in this study’s animal, IBA1-positive and MLANA-positive macrophages could be clearly distinguished (**Figure 5**). This observation indicates that, at least in this primate, intermediate cells and PVMs develop from different precursor cells. Simultaneously, the present study showed that initial integrations of macrophages and intermediate cells proceed in parallel. This observation suggests a cooperative mechanism such as the existence of chemoattractants between the intermediate cells and macrophages in the stria vascularis. Therefore, further studies are required to confirm this conjecture.

Previous studies on rodents and humans have shown that macrophages in the developing cochlea have several subgroups (17). Our study also indicated subgroups in the developing primate animal model cochlea: at least CD163-positive and -negative macrophages (**Figures 7**, **8**), as observed in human fetuses (17). To date, two different lineages of macrophages have been identified in the developing cochlea of rodents: yolk sac macrophages and fetal liver lineages (14). Although we still do not know whether the expression patterns of CD163 in macrophages affect their characteristics as macrophages or indicate lineage differences, our observations showed the possibility of several subgroups in developing cochlear macrophages in this primate animal model.

Antigen presentation plays an essential role in macrophages. In particular, the MHC Class II-mediated presentation of extracellular antigens and subsequent stimulation of CD4^+^ T cells is essential for antigen-specific adaptive immune responses. The present study showed that most IBA1-positive macrophages in the developing cochlea did not express MHC Class II (**Figure 9**). This indicated that the macrophages were not activated for antigen presentation via MHC Class II, at least in the steady state. In contrast, as previously reported in the human cochlea (33), IBA1-positive macrophages in the postnatal cochlea showed MHC Class II expression in common marmosets. Whether these differences in MHC Class II expression between the developing and postnatal cochlea are caused by the maturation status of macrophages or by several mechanisms that downregulate the expression of MHC II (44) has not been clarified. However, our observations suggest that developing cochlear macrophages do not have antigen-presenting activity and may be more engaged in other macrophage functions, such as phagocytosis, which removes cellular debris created during development.

In this study, the distribution of macrophages in the developing cochlea of the common marmoset was examined. Although our previous studies have revealed general cochlear developmental courses of the common marmoset, including hair cells and supporting cells, spiral ganglion neurons, and the stria vascularis (21–24), no detailed examinations from the immunological viewpoint have been performed. In this study, the immunological state of the developing cochlea in the common marmoset was investigated for the first time. Combined with our previous developmental study, we revealed the relationships between generally important developmental stages of the cochlea, such as hair cell differentiation, pruning of spiral ganglion neurons, and the distribution of macrophages (**Figure 10**). This observation is important for understanding the steady immunological state of the developing cochlea in this primate. Recently, cochlear immunological changes have received considerable attention due to their protective and insulating functions (32). Therefore, this study would be useful not only for future studies targeting fetal immunology in the cochlea of this primate, but also for investigations of the damaged cochlea model, including noise-induced hearing loss and ototoxicity in this animal.

In this study, we investigated the distribution of macrophages in the developing cochlea of the common marmoset; however, other immune cells’ distribution and infiltrations have not been examined in this primate. Therefore, future studies can explore this to further understand the immunological steady state of the primate’s cochlear development.

## 5 Conclusion

In the present study, we showed detailed distribution changes of the macrophages in the cochlea using a primate animal model. This observation indicates that most of the changes in the general distribution of macrophages were well preserved between rodents and primates. The distribution changes observed in the common marmoset were also compatible with observations in the human fetus; although, observations in the human fetus are limited. Our observations in this study also revealed several differences between common marmosets and rodents. The study will help researchers understand the primate-specific immunological development of the cochlea and facilitate studies that manipulate the immunological state of the cochlea, especially those focusing on noise trauma or ototoxicity.

## Data availability statement

The original contributions presented in the study are included in the article/supplementary material. Further inquiries can be directed to the corresponding author.

## Ethics statement

The animal study was approved by the Animal Experiment Committee of Keio University. The study was conducted in accordance with the local legislation and institutional requirements.

## Author contributions

MH, TK, MS, TN, NO, HOk, and HOz, conceived and designed the experiments. MH, TK, and MS, wrote the manuscript. MH performed most of the experiments. MH, TK, and MS analyzed the data. All authors read and approved the final version of the manuscript.

## References

[1] Ladrech S Wang J Simonneau L Puel JL Lenoir M. Macrophage contribution to the response of the rat organ of Corti to amikacin. J Neurosci Res (2007) 85:1970–9. doi: 10.1002/jnr.21335 17497672

[2] Hirose K Sato E. Comparative analysis of combination kanamycin-furosemide versus kanamycin alone in the mouse cochlea. Hear Res (2011) 272:108–16. doi: 10.1016/j.heares.2010.10.011 PMC4519356 21044672

[3] Hirose K Discolo CM Keasler JR Ransohoff R. Mononuclear phagocytes migrate into the murine cochlea after acoustic trauma. J Comp Neurol (2005) 489:180–94. doi: 10.1002/cne.20619 15983998

[4] Tornabene SV Sato K Pham L Billings P Keithley EM. Immune cell recruitment following acoustic trauma. Hear Res (2006) 222:115–24. doi: 10.1016/j.heares.2006.09.004 17081714

[5] Tan WJ Thorne PR Vlajkovic SM. Characterisation of cochlear inflammation in mice following acute and chronic noise exposure. Histochem Cell Biol (2016) 146:219–30. doi: 10.1007/s00418-016-1436-5 27109494

[6] Frye MD Yang W Zhang C Xiong B Hu BH. Dynamic activation of basilar membrane macrophages in response to chronic sensory cell degeneration in aging mouse cochleae. Hear Res (2017) 344:125–34. doi: 10.1016/j.heares.2016.11.003 PMC5239751 27837652

[7] Noble K Brown L Elvis P Lang H. Cochlear immune response in presbyacusis: a focus on dysregulation of macrophage activity. J Assoc Res Otolaryngol (2022) 23:1–16. doi: 10.1007/s10162-021-00819-x 34642854 PMC8782976

[8] Hu BH Zhang C Frye MD. Immune cells and non-immune cells with immune function in mamMalian cochleae. Hear Res (2018) 362:14–24. doi: 10.1016/j.heares.2017.12.009 29310977 PMC5911222

[9] Keithley EM. Inner ear immunity. Hear Res (2022) 419:108518. doi: 10.1016/j.heares.2022.108518 35584985

[10] Kaur T Zamani D Tong L Rubel EW Ohlemiller KK Hirose K. Fractalkine Signaling Regulates Macrophage Recruitment into the Cochlea and Promotes the Survival of Spiral Ganglion Neurons after Selective Hair Cell Lesion. J Neurosci (2015) 35:15050–61. doi: 10.1523/JNEUROSCI.2325-15.2015 PMC4642237 26558776

[11] Manickam V Gawande DY Stothert AR Clayman AC Batalkina L Warchol ME. Macrophages promote repair of inner hair cell ribbon synapses following noise-induced cochlear synaptopathy. J Neurosci (2023) 43:2075–89. doi: 10.1523/JNEUROSCI.1273-22.2023 PMC10039750 36810227

[12] Brown LN Xing Y Noble KV Barth JL Panganiban CH Smythe NM. Macrophage-mediated glial cell elimination in the postnatal mouse cochlea. Front Mol Neurosci (2017) 10:407. doi: 10.3389/fnmol.2017.00407 29375297 PMC5770652

[13] Dong Y Zhang C Frye M Yang W Ding D Sharma A. Differential fates of tissue macrophages in the cochlea during postnatal development. Hear Res (2018) 365:110–26. doi: 10.1016/j.heares.2018.05.010 PMC6026078 29804721

[14] Kishimoto I Okano T Nishimura K Motohashi T Omori K. Early development of resident macrophages in the mouse cochlea depends on yolk sac hematopoiesis. Front Neurol (2019) 10:1115. doi: 10.3389/fneur.2019.01115 31695671 PMC6817595

[15] Borse V Kaur T Hinton A Ohlemiller K Warchol ME. Programmed cell death recruits macrophages into the developing mouse cochlea. Front Cell Dev Biol (2021) 9:777836. doi: 10.3389/fcell.2021.777836 34957108 PMC8696258

[16] Kim JH Rodriguez-Vazquez JF Verdugo-Lopez S Cho KH Murakami G Cho BH. Early fetal development of the human cochlea. Anat Rec (Hoboken) (2011) 294:996–1002. doi: 10.1002/ar.21387 21538929

[17] Steinacher C Chacko LJ Liu W Rask-Andersen H Bader W Dudas J. Visualization of macrophage subsets in the development of the fetal human inner ear. Front Immunol (2022) 13:965196. doi: 10.3389/fimmu.2022.965196 36159857 PMC9501668

[18] Lavigne-Rebillard M Bagger-Sjoback D. Development of the human stria vascularis. Hear Res (1992) 64:39–51. doi: 10.1016/0378-5955(92)90166-K 1490899

[19] Locher H Frijns JH Van Iperen L De Groot JC Huisman MA Chuva De Sousa Lopes SM. Neurosensory development and cell fate determination in the human cochlea. Neural Dev (2013) 8:20. doi: 10.1186/1749-8104-8-20 24131517 PMC3854452

[20] Locher H De Groot JC Van Iperen L Huisman MA Frijns JH Chuva De Sousa Lopes SM. Development of the stria vascularis and potassium regulation in the human fetal cochlea: Insights into hereditary sensorineural hearing loss. Dev Neurobiol (2015) 75:1219–40. doi: 10.1002/dneu.22279 PMC5024031 25663387

[21] Hosoya M Fujioka M Murayama AY Okano H Ogawa K. The common marmoset as suitable nonhuman alternative for the analysis of primate cochlear development. FEBS J (2021) 288:325–53. doi: 10.1111/febs.15341 PMC7818239 32323465

[22] Hosoya M Fujioka M Murayama AY Ozawa H Okano H Ogawa K. Neuronal development in the cochlea of a nonhuman primate model, the common marmoset. Dev Neurobiol (2021) 81:905–38. doi: 10.1002/dneu.22850 PMC9298346 34545999

[23] Hosoya M Fujioka M Okahara J Yoshimatsu S Okano H Ozawa H. Early development of the cochlea of the common marmoset, a non-human primate model. Neural Dev (2022) 17:6. doi: 10.1186/s13064-022-00162-8 35524278 PMC9077934

[24] Hosoya M Kitama T Iwabu K Nishiyama T Oishi N Okano H. Development of the stria vascularis in the common marmoset, a primate model. Sci Rep (2022) 12:19811. doi: 10.1038/s41598-022-24380-6 36396805 PMC9672111

[25] Hosoya M Fujioka M Okano H Ozawa H. Mapping of Notch signaling in the developing organ of Corti in common marmosets. Front Neuroanat (2023) 17:1188886. doi: 10.3389/fnana.2023.1188886 37351521 PMC10282542

[26] Sun Z Cheng Z Gong N Xu Z Jin C Wu H. Neural presbycusis at ultra-high frequency in aged common marmosets and rhesus monkeys. Aging (Albany NY) (2021) 13:12587–606. doi: 10.18632/aging.202936 PMC8148503 33909598

[27] Okano H. Current status of and perspectives on the application of marmosets in neurobiology. Annu Rev Neurosci (2021) 44:27–48. doi: 10.1146/annurev-neuro-030520-101844 34236888

[28] Murayama AY Kuwako KI Okahara J Bae BI Okuno M Mashiko H. The polymicrogyria-associated GPR56 promoter preferentially drives gene expression in developing GABAergic neurons in common marmosets. Sci Rep (2020) 10:21516. doi: 10.1038/s41598-020-78608-4 33299078 PMC7726139

[29] Hosoya M Iwabu K Kitama T Nishiyama T Oishi N Okano H. Development of cochlear spiral ligament fibrocytes of the common marmoset, a nonhuman model animal. Sci Rep (2023) 13:11789. doi: 10.1038/s41598-023-39003-x 37479821 PMC10362005

[30] Okano T Nakagawa T Kita T Kada S Yoshimoto M Nakahata T. Bone marrow-derived cells expressing Iba1 are constitutively present as resident tissue macrophages in the mouse cochlea. J Neurosci Res (2008) 86:1758–67. doi: 10.1002/jnr.21625 18253944

[31] Wakabayashi K Fujioka M Kanzaki S Okano HJ Shibata S Yamashita D. Blockade of interleukin-6 signaling suppressed cochlear inflammatory response and improved hearing impairment in noise-damaged mice cochlea. Neurosci Res (2010) 66:345–52. doi: 10.1016/j.neures.2009.12.008 20026135

[32] Liu W Molnar M Garnham C Benav H Rask-Andersen H. Macrophages in the human cochlea: saviors or predators-A study using super-resolution immunohistochemistry. Front Immunol (2018) 9:223. doi: 10.3389/fimmu.2018.00223 29487598 PMC5816790

[33] Liu W Danckwardt-Lilliestrom N Schrott-Fischer A Glueckert R Rask-Andersen H. Distribution of immune cells including macrophages in the human cochlea. Front Neurol (2021) 12:781702. doi: 10.3389/fneur.2021.781702 34880828 PMC8645652

[34] Okayasu T O’malley JT Nadol JB Jr. Prevalence of macrophages within the cochlear vessels following cochlear implantation in the human: an immunohistopathological study using anti-iba1 antibody. Otol Neurotol (2021) 42:e1470–7. doi: 10.1097/MAO.0000000000003312 PMC8595581 34325451

[35] Lee NJ Ha SK Sati P Absinta M Luciano NJ Lefeuvre JA. Spatiotemporal distribution of fibrinogen in marmoset and human inflammatory demyelination. Brain (2018) 141:1637–49. doi: 10.1093/brain/awy082 PMC5972667 29688408

[36] Zhang W Dai M Fridberger A Hassan A Degagne J Neng L. Perivascular-resident macrophage-like melanocytes in the inner ear are essential for the integrity of the intrastrial fluid-blood barrier. Proc Natl Acad Sci USA (2012) 109:10388–93. doi: 10.1073/pnas.1205210109 PMC3387119 22689949

[37] Fabriek BO Dijkstra CD Van Den Berg TK. The macrophage scavenger receptor CD163. Immunobiology (2005) 210:153–60. doi: 10.1016/j.imbio.2005.05.010 16164022

[38] Noble KV Liu T Matthews LJ Schulte BA Lang H. Age-related changes in immune cells of the human cochlea. Front Neurol (2019) 10:895. doi: 10.3389/fneur.2019.00895 31474935 PMC6707808

[39] Rock KL Reits E Neefjes J. Present yourself! By MHC class I and MHC class II molecules. Trends Immunol (2016) 37:724–37. doi: 10.1016/j.it.2016.08.010 PMC5159193 27614798

[40] Yang W Vethanayagam RR Dong Y Cai Q Hu BH. Activation of the antigen presentation function of mononuclear phagocyte populations associated with the basilar membrane of the cochlea after acoustic overstimulation. Neuroscience (2015) 303:1–15. doi: 10.1016/j.neuroscience.2015.05.081 26102003 PMC4532582

[41] Coate TM Scott MK Gurjar M. Current concepts in cochlear ribbon synapse formation. Synapse (2019) 73:e22087. doi: 10.1002/syn.22087 30592086 PMC6573016

[42] Ito T Kurata N Fukunaga Y. Tissue-resident macrophages in the stria vascularis. Front Neurol (2022) 13:818395. doi: 10.3389/fneur.2022.818395 35185769 PMC8850293

[43] Ito T Li X Kurima K Choi BY Wangemann P Griffith AJ. Slc26a4-insufficiency causes fluctuating hearing loss and stria vascularis dysfunction. Neurobiol Dis (2014) 66:53–65. doi: 10.1016/j.nbd.2014.02.002 24561068 PMC3995827

[44] Roche PA Furuta K. The ins and outs of MHC class II-mediated antigen processing and presentation. Nat Rev Immunol (2015) 15:203–16. doi: 10.1038/nri3818 PMC6314495 25720354

